# Toxicity of Nickel Oxide Nanoparticles on a Freshwater Green Algal Strain of* Chlorella vulgaris*

**DOI:** 10.1155/2017/9528180

**Published:** 2017-04-04

**Authors:** Abdallah Oukarroum, Wassila Zaidi, Mahshid Samadani, David Dewez

**Affiliations:** Département de Chimie, Université du Québec à Montréal, C.P. 8888, Succ. Centre-Ville, Montréal, QC, Canada H3C 3P8

## Abstract

A freshwater microalga strain of* Chlorella vulgaris* was used to investigate toxic effects induced by nickel oxide nanoparticles (NiO-NPs) in suspension. Algal cells were exposed during 96 h to 0–100 mg L^−1^ of NiO-NPs and analyzed by flow cytometry. Physicochemical characterization of nanoparticles in tested media showed a soluble fraction (free Ni^2+^) of only 6.42% for 100 mg L^−1^ of NiO-NPs, indicating the low solubility capacity of these NPs. Toxicity analysis showed cellular alterations which were related to NiO-NPs concentration, such as inhibition in cell division (relative cell size and granularity), deterioration of the photosynthetic apparatus (chlorophyll synthesis and photochemical reactions of photosynthesis), and oxidative stress (ROS production). The change in cellular viability demonstrated to be a very sensitive biomarker of NiO-NPs toxicity with EC_50_ of 13.7 mg L^−1^. Analysis by TEM and X-ray confirmed that NiO-NPs were able to cross biological membranes and to accumulate inside algal cells. Therefore, this study provides a characterization of both physicochemical and toxicological properties of NiO-NPs suspensions in tested media. The use of the freshwater strain of* C. vulgaris* demonstrated to be a sensitive bioindicator of NiO-NPs toxicity on the viability of green algae.

## 1. Introduction

During the last 15 years, nanotechnology has been a growing field of innovation worldwide in which several metallic nanoparticles (NPs) have become intensively used in agriculture, industrial products, and medical treatment [1–5]. However, these nanomaterials can be released and transported into the air, soil, and water compartments, representing a risk of danger for environmental quality [6–8]. Therefore, it has been suggested for physicochemical and toxicological properties of nanomaterials to be characterized by several laboratory testing methods permitting environmental risk assessment and safety [9, 10]. Recently, previous toxicological studies on metallic NPs showed toxicity responses directly related to NPs physicochemical properties, such as the shape, the surface area and chemistry, the hydrodynamic size distribution, the concentration, and the solubility [11–14]. In particular, some studies demonstrated that agglomeration of NPs played an important role in determining their cellular toxicity by altering the solubility of NPs [15, 16]. Moreover, others studies showed that agglomeration of NPs was dependent to characteristics of the aqueous media such as pH, ionic strength, and concentration of organic compounds [17, 18]. Therefore, characterization of NPs properties under different environmental conditions represents useful knowledge in toxicity risk assessment and safety management of NPs.

Nickel oxide nanoparticles (NiO-NPs) represent a nanomaterial widely used in the industry for catalysis, alkaline battery cathodes, electrochromic and magnetic materials, pigments in ceramics, and glasses, since possessing unique chemical properties due to its size and morphology when compared to its bulk counterpart (MTI corporation, Manufacturer in Richmond, California, USA). However, it has been reported that NiO-NPs were able to be easily transported into mammalian cellular systems, inducing cytotoxic and genotoxic effects [19]. Moreover, it was observed in sterilized seawater condition performed by Gong et al. (2011) [20] that NiO-NPs (20 nm average size) provoked a severe growth inhibition on a marine microalga strain of* C. vulgaris* when treated with 40–50 mg L^−1^ during 72–120 h of exposure, and this inhibitory effect was caused by cellular morphological alterations such as plasmolysis (leak of cytosol), cytomembrane breakage (detached or degraded plasma membrane), and disorder of thylakoids (grana lamella). In this study, authors focused on the bioremediation ability of marine* C. vulgaris*, suggesting that algal cells were able to increase the agglomeration-deposition capacity of NiO-NPs in seawater condition and also their reduction to zero-valence Ni. However, studies should also be performed to characterize NiO-NPs toxicological properties under freshwater condition in which the salinity does not affect the physicochemical properties of NiO-NPs, such as the agglomeration state. Such knowledge will be necessary in the development of microalgal bioassay of nanotoxicity testing.

Microalgae are widely used in bioassay toxicity testing of aquatic pollutants since they are sensitive organisms with a high capacity of bioaccumulation due to their high surface of contact [21]. In previous toxicological studies, the flow cytometry method has been successfully applied to characterize cellular deterioration caused by the toxicity of metals [22]. Indeed, this method can rapidly provide a multiparametric analysis of algal cell characteristics such as the relative cell size and granularity, cellular viability, chlorophyll *a* fluorescence emission, and other enzymatic activities related to the physiological state of cell [23]. Recently, flow cytometry analysis was successfully used to assess cytotoxicity effects of several metallic nanoparticles such as silica, silver, and copper oxide nanoparticles [24–27]. Therefore, this methodological approach can provide an in-depth investigation to characterize toxic effects of NiO-NPs on the cell physiology of green algae.

In the present study, a freshwater strain of the green algal species* Chlorella vulgaris* was used as a unicellular model organism for the toxicity characterization of NiO-NPs. Algal cells were exposed during 96 hours in order to evaluate the uptake and toxicity impact of NiO-NPs on the entire cellular system by using the flow cytometry method. This work provided valuable results necessary to determine the risk of NiO-NPs toxicity on the viability of this algal strain and therefore its potential use in a bioassay of NiO-NPs toxicity.

## 2. Materials and Method

### 2.1. Algal Culture

The freshwater microalga* C. vulgaris* was obtained from the Canadian Phycological Culture Centre (CPCC, University of Waterloo, Canada). In controlled laboratory conditions, microalga* C. vulgaris* was grown in sterile BG-11 liquid medium [28] at pH 7, under continuous illumination (light intensity of 100 *μ*mol m^−2^ s^−1^ provided by SYLVANIA GRO-LUX Wide Spectrum light F40/GRQ/AQ/WS) and constant temperature of 23 ± 1°C. The stock culture was aerated with bubbling air. Aliquots of algal cells were used as samples for experiments when algal batch-culture was in the exponential growth phase. Algal samples (of 50 mL) having 10^6^ cells mL^−1^ were exposed to 0, 0.1, 1, 10, or 100 mg L^−1^ of NiO-NPs suspensions during 96 h in the condition as described above.

### 2.2. Nickel Oxide Nanoparticles

Nickel oxide nanopowder was purchased from MTI Corporation (Richmond, CA, USA). According to the manufacturer, the diameter of NiO-NPs was 30 nm, purity was 99.5%, and the specific surface area was 50–80 m^2^ g^−1^. In this study, NiO-NPs size distribution in BG-11 culture medium was determined by dynamic light scattering (DLS) with a ZetaPlus Particle Sizer (Brookhaven Instruments Corporation, USA) using a 90Plus Particles Sizing Software (Vers. 4.20). A stock solution of 100 mg L^−1^ of NiO-NPs was prepared, and the suspension was homogenized before use during 5 min by ultrasonication. To determine the solubility of NiO-NPs, suspensions of 0–100 mg L^−1^ without alga cells were prepared and incubated during 96 h in the same condition as described above for algal culture. Then, NiO-NPs suspensions were centrifuged at 12,000*g* for 30 min. The supernatant was removed with care, and the quantification of Ni in 10% HNO_3_ was done by Atomic Absorption Spectrometry using a Varian SpectrAA 220 FS system (detection limits for Ni: 0.06–3000 ppm).

### 2.3. Determination of Total Chlorophyll

Chlorophyll content was extracted from 1 mL of algal sample in 100% methanol. The extract was heated at 65°C for 10 min and pigments were separated by centrifugation. Quantitative determination of chlorophyll content (Chl* a* + Chl* b*) was performed using a UV/Vis spectrometer (Lambda 40, Perkin Elmer) and the following equations [29]:

(1) Chl  aμg mL−1=12.25A663–A750−2.79A647–A750,Chl  bμg mL−1=21.50A647–A750−5.10A663–A750.

### 2.4. Flow Cytometry Approach

A FACScan flow cytometer (Becton Dickinson Instruments) was used to determine specific cellular parameters. The forward light scatter signal (FSC) was used as an indication of the relative size of the cell [30], the cellular granularity was determined by using the side light scatter signal (SSC), and the chlorophyll *a* fluorescence was measured. Viable cells were estimated by using the molecular probe fluorescein diacetate (FDA). FDA is a nonpolar ester compound which passes through cell membranes. Once inside the cell, esterases (active enzymes present only in viable cells) will hydrolyze FDA into fluorescein, a compound emitting fluorescence under UV illumination [31]. Moreover, the production of reactive oxygen species (ROS) in algal cells was determined by using the permeable dye indicator 2′,7′-dichlorodihydrofluorescein diacetate (H_2_DCFDA). Both FDA and H_2_DCFDA stock solutions (of 10 mM) were prepared in ethanol in the dark. At the end of the 96 h of exposure, algal samples of 1 mL from experimental cultures were exposed during 30 min to 10 *μ*M of FDA or H_2_DCFDA in the dark and the fluorescence emission at 530 nm was measured. This fluorescence emission was indicative of the level of viable cells or intracellular ROS, respectively. The mean of fluorescence for any given algal cell population was provided by Cyflogic software.

### 2.5. Nickel Content in Algal Biomass

At 96 h of treatment, algal cells were separated from NiO-NPs by centrifugation using different sucrose concentrations [27]. These sucrose solutions of 20, 40, 60, 80, 100, and 120% were prepared in BG-11 medium. Starting from the highest to the lowest sucrose concentration, 3 mL of each sucrose solution was gently placed in a glass tube inclined at 30° angle. 50 and 100 mg L^−1^ of NiO-NPs-treated cultures were centrifuged and the pellet was slowly placed on the top of the sucrose gradient. Tubes were centrifuged at 1000 rpm during 20 min and a clear separation between algal cells and NiO-NPs was observed. The algal layer forming a pellet at the bottom of the tube was recuperated using a glass Pasteur pipette and filtered on a 0.45 *μ*m filter previously dried and weighted. To remove Ni that might be bound to the cell surface or the filter, 3 × 10 mL of 10 mM ethylene diamine tetraacetic acid in BG-11 medium was passed through the filter. Filters were dried at 95°C for 24 h, weighted to calculate algal dry mass, and then placed in acid-washed glass tubes in which 4 mL HNO_3_ and 500 *μ*L H_2_O_2_ were added progressively. Samples were digested during 48 h at room temperature before being diluted to 20% HNO_3_ in Nanopure purified water for the quantification of Ni by atomic absorption spectrometry.

### 2.6. Transmission Electronic Microscopy (TEM) Analysis

At 96 h of treatment, samples of 50 mL from control and 100 mg L^−1^ NPs-treated cultures were centrifuged. The supernatant was discarded and the pellet was washed with 500 *μ*L of washing buffer (0.1 M cacodylate, 0.1% CaCl_2_, pH 7.2) for 10 s. The washing buffer was removed and 500 *μ*L of fixation buffer (2.5% glutaraldehyde in 0.1 M washing buffer) was added. Cells were fixed overnight at 4°C. Then, the pellet was washed three times during 10 min with the washing buffer and stained with osmium tetroxide (1% OsO_4_, 1.5% KFeCN in water) during 2 h at 4°C. After osmium tetroxide treatment, the pellet was washed with Nanopure purified water (3 × 10 min), dehydrated with increasing concentrations of acetone (30, 50, 70, 80, 90, and 100% acetone, 3 × 10 min each). Dehydrated samples were infiltrated with epon at room temperature with increasing concentrations of epon in acetone: 1 : 1 (overnight), 2 : 1 (4 h), and 3 : 1 (overnight). Then, the pellet was placed in pure epon, left 4 h under vacuum, and then heated 48 h at 58°C. Hardened pellets were cut into 0.6 *μ*m thick slices and placed on a gold grid for TEM analysis. Samples were visualized with a FEI Tecnai 12 120 kV microscope and pictures taken with a Gatan 792 Bioscan 1 × 1 k Wide Angle Multiscan CCD camera. The elemental nature of the metallic agglomerates found inside the cell was determined by energy-dispersive X-ray spectroscopy (EDX) done with the same section thickness placed onto a carbon-coated grid using a Philips CM200 200 kV TEM equipped with a Gatan Ultrascan 1000 2 k × 2 k CCD Camera System Model 895 and a EDAX Genesis EDS.

### 2.7. Data Analysis and Statistics

Cytometry analysis was done in each sample for 10^4^ events (cells). The mean of fluorescence for any given population was provided by Cyflogic software. Means were determined for each treatment. Significant differences between control and treated samples were determined by using a multiple comparison Tukey's test, where *p* value less than 0.05 was considered to be significantly different. The median effective concentration given at 50% (EC_50_) was obtained by the Log inhibitor concentration versus the response fitting the toxicity data using Prism software.

## 3. Results

### 3.1. Physicochemical Characterization of NiO-NPs

**Figure 1:**
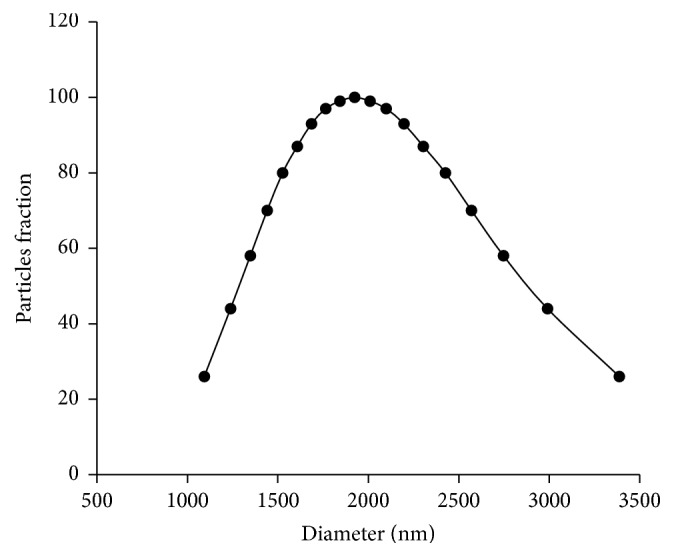
Log-normal of NiO-NPs size distribution suspended and incubated in BG-11 medium for 96 h. A stock suspension of 100 mg L^−1^ was prepared and sonicated before use during 5 min using an ultrasonicator.

Hydrodynamic size distribution, surface charge, and solubility of NiO-NPs were determined in the BG-11 medium at 96 h of incubation. Hydrodynamic size distribution was estimated based on dynamic light scattering of NiO-NPs suspensions at the highest tested concentration (100 mg L^−1^). Measurements showed a large particle size distribution having a median of 2000 nm, indicating the formation of agglomerates having sizes of 1100–3500 nm (Figure 1). Moreover, the solubility of NiO-NPs suspensions was quantified under this experimental condition. Results indicated that the concentration of total soluble Ni increased when the exposure concentration of NiO-NPs suspensions increased (Table 1). However, the proportion of the soluble fraction of Ni was low, representing 8 and 6.4%, respectively, for 10 mg L^−1^ and 100 mg L^−1^ of NiO-NPs suspended in the BG-11 medium.

### 3.2. Flow Cytometry Analysis

**Figure 2:**
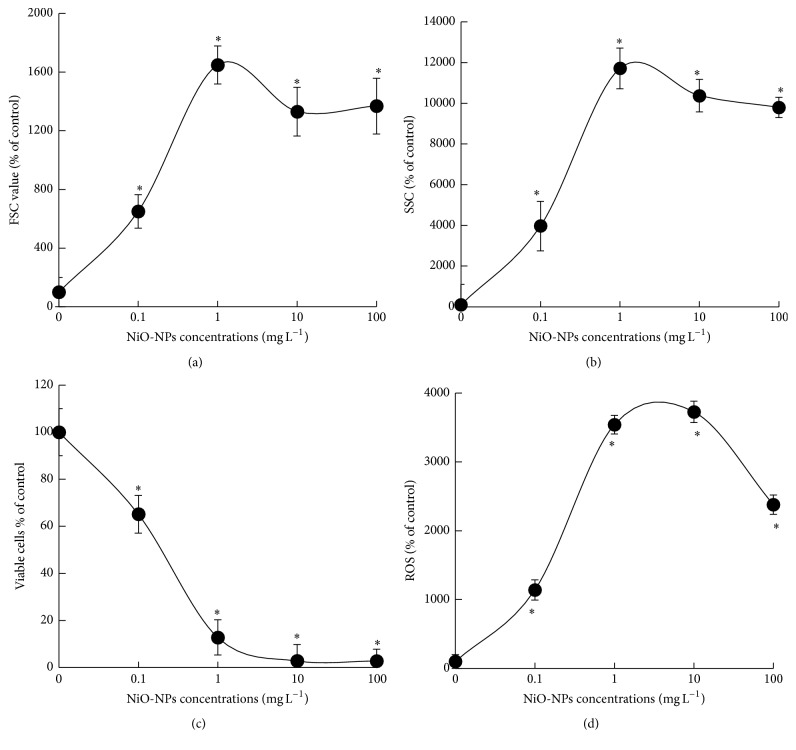
Change of relative cell size (a), cellular granularity (b), viable cells (c), and production of intracellular reactive oxygen species (d) in algal cells of* C. vulgaris* exposed during 96 h to different concentrations of NiO-NPs suspensions. The asterisk (*∗*) indicated significant difference between control and treated samples for *p* < 0.05.

When algal cells of* C. vulgaris* were exposed during 96 h to different concentrations of NiO-NPs, cytotoxicity effects were investigated by evaluating cellular biomarkers such as the relative cell size, granularity, cellular viability, and induction of ROS generation (Figure 2). Obtained results indicated that NiO-NPs cytotoxic effects provoked a strong significant increase in the relative size of* C. vulgaris* cells which was directly related to the increasing concentration of NiO-NPs suspensions (Figure 2(a)). Compared to the control sample, the relative cell size of treated algal cells to 1 mg L^−1^ of NiO-NPs did increase significantly to the highest level (*p* < 0.05). Furthermore, the change in cellular granularity presented similar tendency as the relative cell size in relation to the concentration of NiO-NPs suspensions (Figure 2(b)). The cellular granularity increased significantly (*p* < 0.05) dependent on NiO-NPs concentration, and the highest increase was observed when* C. vulgaris* cells were exposed to 1 mg L^−1^ of NiO-NPs. Additionally, the effect of different concentrations of NiO-NPs suspensions was investigated on the viability of algal cells which was presented in Figure 2(c). Our results showed that the viability of treated algal cells decreased significantly (*p* < 0.05) compared to control, which was related to the increasing concentration of NiO-NPs. At 96 h of NiO-NPs exposure, the proportion of viable cells decreased significantly compared to control (*p* < 0.05) by 35 and 87%, respectively, for treatments of 0.1 and 1 mg L^−1^, and also by 97% for both treatments of 10 and 100 mg L^−1^ of NiO-NPs (Figure 2(c)). Therefore, the evaluation of the EC_50_ based on the change of viable cells indicated a concentration value of 13.74 (±0.94) mg L^−1^. Moreover, the induction of intracellular ROS production following the exposure of algal cells to NiO-NPs suspensions was probed by fluorescence emission (Figure 2(d)). Based on obtained results, we found a significant increase in ROS production (*p* < 0.05) in treated algal cells when compared to control, and this change was dependent on the concentration of NiO-NPs. When algal cells were exposed during 96 h to 10 mg L^−1^ of NiO-NPs, the production of ROS showed the strongest level, which was 37 times higher than the control level (Figure 2(d)). However, at 100 mg L^−1^ of NiO-NPs, the production of ROS showed a decrease probably due to a strong accumulation of dead cells.

### 3.3. Chlorophyll Content and Chlorophyll a Fluorescence

**Figure 3:**
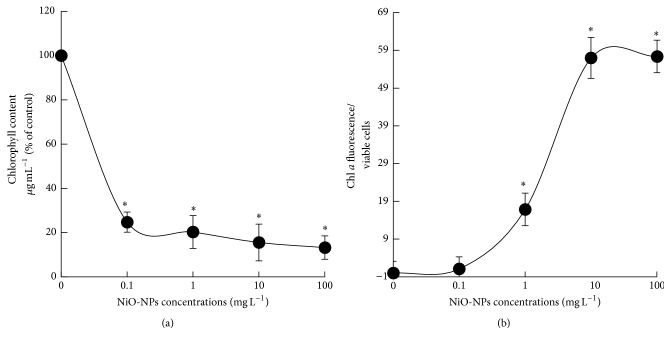
Change of the total chlorophyll content (a) and the emission of chlorophyll *a* fluorescence (b) in algal cells of* C. vulgaris* exposed during 96 h to different concentrations of NiO-NPs suspensions. The asterisk (*∗*) indicated significant difference between control and treated samples for *p* < 0.05.

When algal cells of* C. vulgaris* were exposed to NiO-NPs suspensions from 0.01 to 100 mg L^−1^ during 96 h, a strong reduction in the total chlorophyll content was observed (Figure 3(a)), indicating an inhibition in chlorophyll synthesis. Total chlorophyll content decreased by 75, 80, 85, and 87% compared to control (*p* < 0.05), respectively, for treatment concentrations of 0.1, 1, 10, and 100 mg L^−1^ of NiO-NPs. Moreover, the analysis of chlorophyll *a* fluorescence emission was used to inform on the functional state of the photosynthetic apparatus. Obtained results showed that fluorescence intensity per viable cells strongly increased in comparison to control (*p* < 0.05) when algal cells of* C. vulgaris* were exposed to 1–100 mg L^−1^ of NiO-NPs (Figure 3(b)), indicating significant inhibition in photochemical reactions of photosynthesis.

### 3.4. Intracellular Nickel and NiO-NPs

**Figure 4:**
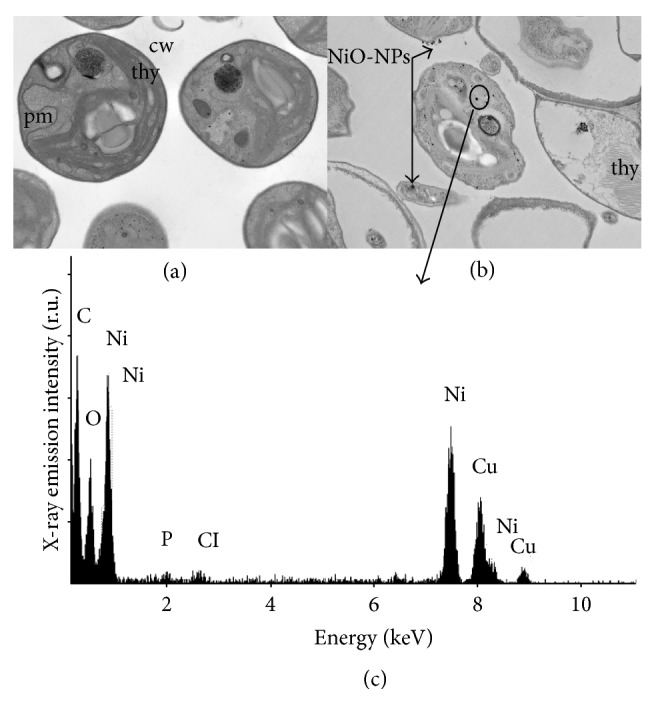
TEM images of* C. vulgaris* control cells (a) and cells treated during 96 h with 100 mg L^−1^ of NiO-NPs (b). In the lower part (c), energy-dispersive X-ray spectroscopy analysis of metals found in the cytoplasm of* C. vulgaris* cells treated with 100 mg L^−1^ of NiO-NPs. cw, cell wall; thy, thylakoids; pm, plasma membrane.

When algal cells were exposed during 96 h to 100 mg L^−1^ of NiO-NPs suspensions, the uptake of Ni was measured by a quantitative analysis using atomic absorption spectrometry. Obtained results showed that the total Ni content in algal biomass was of 11.8 mg g^−1^ of dry weight. Furthermore, TEM microscopy was used to compare control cells with treated cells (Figures 4(a) and 4(b)) to determine if NiO-NPs penetrated into algal cells. Interestingly, NiO-NPs agglomerates (black dots) were observed in the cytoplasm of algal treated cells and cellular ultrastructures showed clear morphological alterations such as the loss of membrane integrity (Figure 4(b)). Moreover, energy-dispersive X-ray spectroscopy confirmed the presence of Ni agglomerates inside algal cells (Figure 4). The presence of X-ray peaks representing C, P, and Cl was attributed to biological components of the cell.

## 4. Discussion

In this study, physicochemical properties of NiO-NPs suspensions were characterized in sterilized freshwater BG-11 medium, since it permitted better understanding of NP's behavior in this aqueous solution and to determine NP's mechanism of toxicity on algal cells. When NiO-NPs suspensions were incubated during 96 h, NPs formed several agglomerates in algal culture medium, as indicated by the distribution of the hydrodynamic particles size diameter. These agglomerations were caused by both the neutral pH and the ionic strength of the media, as it was previously reported for different nanomaterials [32–34]. This distribution of hydrodynamic particles size was found to be stable during the entire experimental exposure. Moreover, the formation of NiO-NPs agglomerates may explain the low solubility property of theses NPs in freshwater BG-11 medium during the incubation of 96 h. However, these agglomerates can also be adsorbed at the surface of algal cells entrapping them. This would cause a reduction of the availability of light and nutrients necessary for photosynthesis and therefore induce an inhibition of cellular division [13]. In a previous study [20], authors showed that algal cells of a marine strain of* C. vulgaris* in sterilized seawater f/2 medium did form agglomerations with NiO-NPs suspensions, and such complexes cell-NPs were harmful to the growth of algal cells. In this study, it is most likely that the salinity effect increased the formation of NPs agglomerations with algal cells by changing their surface charge. However, similar results were also found in freshwater condition when algal cells of* P. subcapitata*,* Chlorella* sp., and* Scenedesmus* sp. were exposed to SiO_2_ and TiO_2_ NPs, which were able to be directly adsorbed at the surface of the cell wall [35, 36].

In order to have an in-depth understanding of the toxicity impact of NiO-NPs, cellular alterations of* C. vulgaris* were investigated when algal cells were exposed during 96 h to 0.1–100 mg L^−1^ of NiO-NPs suspensions. According to obtained results, NiO-NPs were able to cause a significant toxicity impact on algal cells, which increased in direct relation to the increasing exposure concentration of nanoparticle's suspension. In fact, relative cell size and cellular granularity of* C. vulgaris* increased strongly in the presence of NiO-NPs suspension, indicating that this NP caused an alteration in the cellular division processes. Indeed, such cellular effect was previously observed in algal cells of* Chlorella kessleri* exposed to silica NPs, and it was suggested to be due to the presence of nanostructures obstructing cell division processes [24]. Furthermore, the toxicity impact of NiO-NPs was significantly indicated by the decrease in viable cells of* C. vulgaris*, which was caused by a total impairment of enzymatic activities and/or the loss of cell membrane integrity. Based on these results, estimated EC_50_ on cellular viability was of 13.7 mg L^−1^, which was much lower than NiO-NPs EC_50_ based on the inhibitory growth rate of 32.3 mg L^−1^, as presented previously [20]. Therefore, it clearly appeared under freshwater experimental conditions that our strain of* C. vulgaris* was more sensitive than the marine strain in this previous study to the toxic effects of NiO-NPs suspension. Indeed, our results demonstrated significant cellular alterations on* C. vulgaris*, which was already induced at low exposure concentrations of NiO-NPs suspension (0.1 and 1 mg L^−1^). On the other hand, the mean solubility of NiO-NPs was stronger in freshwater BG-11 medium (7%) than in seawater f/2 medium (0.14%). Therefore, the bioavailability of toxic Ni^2+^ to algal cells was higher in freshwater BG-11 medium. However, it was difficult to determine here if NiO-NPs were more bioavailable to algal cells in freshwater BG-11 medium in comparison to seawater f/2 medium. Nevertheless, the change in cellular viability of alga* C. vulgaris* demonstrated to be a very sensitive biomarker of NiO-NPs toxicity potential in our freshwater toxicological condition.

Based on our results, we demonstrated that the loss in cellular viability of* C. vulgaris* was caused by several cellular alterations, such as the inhibition in cellular division processes (relative cell size and granularity), the deterioration of photosynthetic apparatus (chlorophyll synthesis and photochemical reactions of photosynthesis), and the generation of ROS. Indeed, we suggest that the exposure to NiO-NPs suspensions induced a strong oxidative stress effect in algal cells of* C. vulgaris*, causing the deterioration of photosynthetic and enzymatic systems permitting cellular growth. Our results were consistent with previous studies suggesting that the induced cytotoxicity effect of several engineered-NPs was directly related to the generation of ROS causing a strong oxidative stress [13, 27, 37–39].

In our study, our results suggest a complex mechanism of toxicity of NiO-NPs on algal cells of* C. vulgaris*. The uptake by algal cells of soluble Ni^2+^ released from NiO-NPs represents one possible mechanism of toxicity. Indeed, it was previously proposed that the solubilization of NPs causing the release of toxic metal ions was considered until recently to be the most common mechanism of toxicity on aquatic microorganisms for several types of metallic NPs [40]. However, the low solubility characteristic of NiO-NPs has been reported to be classified as almost an insoluble material [41]. Moreover, based on the strong induced oxidative stress effect in algal cells, it is most likely that the release of free metal ions from NPs was not the only contributor to the toxicity impact in the algal cellular system. Recently, it was also suggested that the toxicity mechanism of NPs could be directly related to their penetration into cells of aquatic organisms [42]. In our study, we identified the bioaccumulated Ni in algal biomass. The analysis by TEM and X-ray confirmed that NiO-NPs were able to cross the biological membrane and to accumulate inside the cell. Moreover, it was possible that the solubilization of bioaccumulated NiO-NPs happened inside the cell, possessing a more acidic pH environment than the culture media, and then the released Ni^2+^ contributed to the toxicity impact in the algal cellular system. Such hypothesis was previously proposed for Ag-NPs toxicity mechanism in algal cells [43]. However, more studies need to be performed with new advanced analytical technology in order to clarify the toxicity mechanism from the solubilization of bioaccumulated metallic NPs.

## 5. Conclusion

This study permitted determination of the potential source of toxicity of NiO-NPs suspensions on algal cells of the freshwater strain* C. vulgaris*, which demonstrated to be a valuable toxicity bioindicator of NiO-NPs suspensions. The exposure effect of NiO-NPs suspensions with algal cells caused the deterioration of several cellular characteristics. Therefore, this bioassay permitted characterizing some toxicological properties of NiO-NPs suspensions, permitting better understanding of the toxicity risk of NiO-NPs on the viability of green algae.

## Figures and Tables

**Table 1:** Soluble fraction of nickel (mg L^−1^) released from NiO-NPs in BG-11 culture medium at pH 7.

[NiO-NPs]	[Ni^2+^]
mg L^−1^	mg L^−1^
0.1	n/a
1	n/a
10	0.80 ± 0.05
100	6.42 ± 0.6

n/a: not detected.

## Abbreviations

NPs: Nanoparticles.
